# 3D Spatial Distribution of Nanoparticles in Mice Brain Metastases by X-ray Phase-Contrast Tomography

**DOI:** 10.3389/fonc.2021.554668

**Published:** 2021-05-25

**Authors:** Elena Longo, Lucie Sancey, Alessia Cedola, Emmanuel L. Barbier, Alberto Bravin, Francesco Brun, Inna Bukreeva, Michela Fratini, Lorenzo Massimi, Imke Greving, Geraldine Le Duc, Olivier Tillement, Ombeline De La Rochefoucauld, Philippe Zeitoun

**Affiliations:** ^1^ Helmholtz-Zentrum Hereon, Institute of Materials Physics, Geesthacht, Germany; ^2^ Laboratoire d’Optique Appliquée UMR7639, ENSTA-CNRS-Ecole Polytechnique IP Paris, Palaiseau, France; ^3^ Institute for Advanced Biosciences U1209 UMR5309 UGA, Allée des Alpes-Site Santé, La Tronche, France; ^4^ Institute of Nanotechnology—CNR, Rome-Unit, Rome, Italy; ^5^ Univ. Grenoble Alpes, Inserm, U1216, Grenoble Institut Neurosciences, GIN, Grenoble, France; ^6^ European Synchrotron Radiation Facility, Grenoble, France; ^7^ P. N. Lebedev Physical Institute, RAS, Moscow, Russia; ^8^ IRCCS Santa Lucia Foundation, Rome, Italy; ^9^ Department of Medical Physics and Biomedical Engineering, University College London, London, United Kingdom; ^10^ NH TherAguix, Villeurbanne, France; ^11^ Institut lumière-matière, UMR5306, Université Claude Bernard Lyon1-CNRS, Université de Lyon, Villeurbanne, France; ^12^ Imagine Optic, Talence, France

**Keywords:** 3D visualization, melanoma metastases, brain, nanoparticles, synchrotron radiation, X-ray phase-contrast tomography

## Abstract

Characterizing nanoparticles (NPs) distribution in multiple and complex metastases is of fundamental relevance for the development of radiological protocols based on NPs administration. In the literature, there have been advances in monitoring NPs in tissues. However, the lack of 3D information is still an issue. X-ray phase-contrast tomography (XPCT) is a 3D label-free, non-invasive and multi-scale approach allowing imaging anatomical details with high spatial and contrast resolutions. Here an XPCT qualitative study on NPs distribution in a mouse brain model of melanoma metastases injected with gadolinium-based NPs for theranostics is presented. For the first time, XPCT images show the NPs uptake at micrometer resolution over the full brain. Our results revealed a heterogeneous distribution of the NPs inside the melanoma metastases, bridging the gap in spatial resolution between magnetic resonance imaging and histology. Our findings demonstrated that XPCT is a reliable technique for NPs detection and can be considered as an emerging method for the study of NPs distribution in organs.

## Introduction

Metal-based nanoparticles (NPs) demonstrated the interesting capability to increase the radiosensitization of tumors by causing local dose-enhancement (1–6). Thanks to the high-atomic number atoms composing their inner core, NPs produce a shower of Auger electrons following irradiation. In turn, Auger electrons produce reactive oxygen species inducing damage to tumor cells contributing to tumor eradication (7–9). In order to predict therapeutic effects accurately, it is crucial to know the NPs distribution at multiple length scales from cell to the full organ. Furthermore, the possibility of mapping the NPs can permit to evaluate the homogeneity of distribution over the targeted tumor volume (10). Currently, NPs detection in biologic organs is based on the state-of-art imaging techniques such as magnetic resonance imaging (MRI) (11), positron emission tomography (PET) (12), spectral photon counting computed tomography (SPCCT) (13), transmission electron microscopy (TEM) (14), laser-induced breakdown spectroscopy (LIBS) (15), and optical microscopy (16). MRI, PET, SPCCT are 3D techniques able to show the tumor targeting and qualifying the NPs uptake in tumors, covering a spatial resolution range from few mm down to ≤ 0.1 mm (17, 18). SPCCT is specifically advantageous for discriminating and quantifying the signal of the contrast agents relying on K-edge imaging as shown in recent *ex-vivo* and *in-vivo* studies (19–22). Often however the visualization of biological tissues is limited in SPCCT even if the contrast agents concentration is sufficiently high. LIBS and TEM are pseudo-3D techniques and may reach 10 µm down to a few tens of nm spatial resolution, respectively (23, 24). Both techniques are able to detect specific chemical elements contained in NPs. However, they require a complex sample preparation based on physical sample sectioning and staining for enhancing tissue visualization in the case of TEM. For detecting NPs by light microscopy, it is necessary to craft fluorophores on NPs surface: this may lead to the eventual alteration of NPs penetration and aggregation dynamics (25, 26). The absence of non-destructive and non-invasive 3D imaging methods at a median scale resolution ranging between ≤1 and 100 microns is an important limitation for imaging the distribution of NPs inside tumors. This information would be crucial in optimizing radiotherapy protocols based on NPs administration. Synchrotron-based XPCT is a suitable imaging tool for accessing this intermediate resolution range. Phase-contrast is an added value to the tomographic approach. Since imaging biological samples suffer from poor contrast properties in the X-rays regime, imaging using phase-contrast helps to discriminate those features inaccessible in absorption-contrast (27–29). XPCT enables the fast visualization of a whole organ while visualizing small details down to few micrometers (30–32). This can be achieved without the addition of any exogenous substance and with the advantage of preserving the sample integrity (33–35). Moreover, past pre-clinical studies have shown that XPCT is able to detect tumors in mice brains (36–38). Mapping the NPs distribution in brain tumors is today a new frontier of the XPCT. This article shows a proof-of-concept study of NPs tracking in melanoma brain metastases revealing unprecedented images about the NPs location in tumors. Melanoma is one of the most aggressive skin cancers, known for its high resistance to drugs, to radiotherapy treatments and its strong tendency to metastasize (39). In 60% of the cases, melanoma patients develop multiple metastases in the central nervous system leading to mortality (40, 41). Biocompatible nanotechnologies, based on the delivery of non-toxic metal-loaded NPs able to permeate the blood-brain barrier (BBB), could provide a higher patient survival by enhanced radiation therapy. This XPCT work aims at resolving the NPs distribution [here ultra-small gadolinium (Gd)-based NPs (42)] inside a mouse brain model of multiple melanoma metastatic lesions. The NPs tumor targeting is here visualized in 3D over the full brain model. This first experiment demonstrates that XPCT is a suitable technique for estimating the homogeneity of the NPs uptake in multiple metastases. The same brain was also investigated by MRI and histology (3). XPCT enabled to simultaneously visualize the NPs and the brain structures, thus allowing to monitor and establish the correlation between the NPs and brain tissues. Thanks to its higher spatial resolution, the XPCT findings resolve more distinctly the NPs repartition in tumors adding more details to the conventional MRI. In addition, thanks to its contrast resolution, this XPCT work sheds light on the complex internal structure of the metastases grown in the mouse brain. The XPCT ability in detecting the melanoma has been validated also by conventional histology. Therefore, the herewith presented imaging technique reveals to be attractive for complementing the techniques in use for monitoring the NP distribution at the pre-clinical phase.

## Methods

### Nanoparticle Specifications

Gadolinium-based nanoparticles (NPs) namely AGuIX (1,4,7,10-tetra-azacyclododecane-1-glutaric anhydride- 4,7,10-triacetic acid)-Gd^3+^ are comprised of a polysiloxane inorganic matrix surrounded by gadolinium chelates (DOTAGA) covalently grafted to the polysiloxane core (42, 43). AGuIX exhibits a sub-5 nm size with a hydrodynamic diameter of approximately 3 nm (42). In order to obtain such ultra-small NPs, it has been established a new synthesis protocol based on DOTAGA, a cyclic ligand entrapping the Gd^3+^. The AGuIX complexation constant resulted to be 24.78, in good agreement with that of the commercial agent DOTAREM^®^ (Guerbet LLC, Aulnay-sous-Bois, France), 25.58. AGuIX exhibits 10 chelates per nanoparticle and an approximate mass of 10 kDa. The nanoparticles are almost spherical, with a zeta potential of 9 ± 5 mV at physiological pH. The purification occurs *via* dialysis. It has been demonstrated that AGuIX is an effective MRI-positive contrast agent with a longitudinal relaxivity (*r*_1_) up to three times higher than that of DOTAREM, e.g., 11.4 mmol^−1^ s^−1^ and a ratio transverse relaxivity (*r*_2_)/*r*_1_ of 1.14 at 1.4 T (2). This compound, AGuIX, is also produced for human use. The synthesis and purification permit to remove free gadolinium atoms. AGuIX is presently used in phase I to II clinical trials (NCT02820454, NCT03818386, and NCT03308604) as visible in www.clinicaltrials.gov.

In previous mice experiments, these nanoparticles showed a half-life time in blood of 21.6 min and an excellent fast renal elimination reaching a maximum of renal accumulation 4 h after injection, thus limiting most of toxicity consequence in mice body (26).

### Sample Preparation

All animal studies and experiments were approved by the French Ministry of Agriculture and carried out in accordance with the official regulation of the French Ministry of Agriculture after approval by the local Ethical Committee (from Lyon and Grenoble’s Universities, no 380922). All efforts were made to minimize the number of animals used and their suffering due to the experimental procedure. All mice were housed in a specific pathogen-free (SPF) environment. As described in (3), B16F10 cells (# CRL-6475, LGC Promochem, Molsheim France), which are metastatic melanoma cells (50,000 cells) from mouse origin, were implanted in the brain of a mouse 6-week-old C57BL/6J (Janvier, France). The main tumor developed at the injection site and the multiple metastases diffused in the brain. The mouse was euthanized 1 h after the intravenous injection of AGuIX-NPs, 200 µl at 10 mM. The brain (approximately 1 × 1 × 1.5 cm) was removed and fixed in PFA 4%, 1 h at 4°C, before storing in PBS at 4°C. The brain of a control mouse and a sub-cutaneous melanoma sample were similarly sampled for comparison. The samples were embedded in agar agar gel and included within Eppendorf tubes for the XPCT experiment. The embedding in agar agar keeps the sample hydrated and prevents potential sample drifts (33).

### In-Line X-Ray Phase-Contrast Tomography (XPCT) Setup

Mouse brain-bearing multiple melanoma metastases, a melanoma tumor and a healthy mouse brain were imaged post-mortem at the biomedical beamline ID17 of the European Synchrotron Radiation Facility (ESRF) in Grenoble, France. Synchrotron radiation with a quasi-monochromatic X-ray parallel beam was used to illuminate the brains and achieve high-resolution images. The energy was set at 51.5 keV, above Gd K-edge in order to take advantage of the great contrast properties provided by Gd in respect with gray matter. The samples contained in Eppendorf tubes were placed on a high-resolution rotation stage. The XPCT experiment was carried out according to the “in-line” geometry (28). The propagation distance between the sample and the detector was set at 2.3 m. The projection images were recorded by a CMOS camera connected to an optical system having a final pixel size of 3 µm (44). Because the field of view of the camera was 7 mm × 6.4 mm, the entire brains were measured in half-acquisition mode over 360° and virtually divided in two pieces along the vertical axis. A tomographic scan made up of 4,000 projections was performed for each piece of brain with an acquisition time of 0.07 s for a single-image.

### Data Processing and Computational Platform

The full data sets were reconstructed using the SYRMEP TomoProject software (45). The phase was retrieved using Paganin’s algorithm (46), and the slices were reconstructed by applying the Filtered Back Projection algorithm. The reconstructed images exhibit an effective pixel size equal to 3 µm and a spatial resolution equal to 6 µm. The images were further analyzed using FIJI image-processing package, through which the images of the projections of maximum and minimum intensity values were created and combined with each other. Maximum and minimum intensities are known visualization techniques that compress a 3D volume into a 2D image (47). An image of maximum intensity projection is determined by the brightest pixel intensities along a projection path; in our experiment it corresponds to the densest objects. Since Gd is a high-Z metal characterized by a high-attenuation coefficient, with respect to brain tissue and NPs tend to form clusters of different sizes and shapes after the injection, NPs appear like very dense white objects in X-ray tomography images. An image of minimum intensity projection displays the minimum pixel values encountered over a path length, instead. It allows knowing where the areas with low attenuation coefficient are, such as empty vessels or melanin pigments. To avoid intensity fluctuations over the projection rays, the methods of projection of maximum and minimum intensity are generally applied by superimposing a limited number of slices and not to the entire data set (47). By summing these two different ways of visualization, it is possible to get 2D images showing limited volume reconstructions. The open-source software VolView (https://www.kitware.com/volview/) was used to create surface-rendered images and to segment 3D volumes in false color scale, by establishing a transfer function between the gray levels of the image and a color map. The CNR was estimated with reference to the tumor/metastases regions loaded with NPs and to the healthy brain tissue without NPs. The calculations were done using the equation described in (48):

(1) $$CNR=\frac{I_{NPs}-I_{BG}}{{\left[\left(1/2)(\sigma_{NPs}^{2}+\sigma_{BG}^{2}\right)\right]}^{1/2}}$$

*I_NPs_* and *I_BG_* are the mean gray values of a 5 × 5 pixels region of interest (ROI) selected within the tumors with NPs clusters and within the background, while *σ_NPs_* and *σ_BG_* are the standard deviations referred to the two different ROIs. The same calculation was repeated considering the mean gray values and the standard deviation of a 5 × 5 pixels ROI selected in the brain tissue without NPs. As reported by (49), this formula was proven to be efficient in evaluating the CNR for tissues.

### Magnetic Resonance Imaging

MRI was performed on a 9.4 T scanner (Biospec 94/20 AV III HD, Bruker, Germany—Grenoble MRI facility IRMaGE) equipped with a 12-cm inner diameter actively shielded gradient insert (640 mT/m in 120 µm). Actively, decoupled volume and surface coils were used for excitation and reception, respectively (Bruker, Germany). The 3D T1 MRI sequence used the following settings: TR/TE = 31/6.5 ms, flip angle = 20°, acquisition matrix = 184 × 184 × 184, field of view= 11 × 11 × 11 mm^3^, resolution = 60 × 60 × 60 µm^3^.

### Histology

The brain was dehydrated and paraffin-embedded for long term storage before sectioning. The 7-µm slices were deparaffined, re-hydrated, and stained with hematoxylin/eosin (HE) before mounting with Pertex mounting medium (Leica, France). The slices were imaged using a full field epilfluorescence microscope equipped with color camera, and a × 2.5 magnification [Axio Imager M2 (Zeiss, Germany)].

## Results

**Figure 1:**
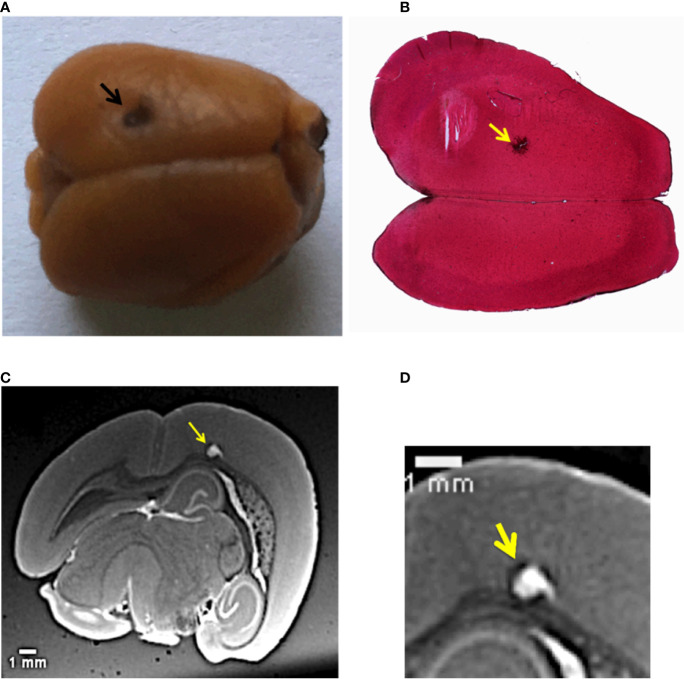
Multi-modal images of the melanoma primary tumor. **(A)** Image of the mouse brain showing the primary tumor. The black dot on the left upper hemisphere, indicated by the black arrow, represents the injection point of the tumor cells. **(B)** Histology image of the brain with a ×2.5 objective. The brain slice was stained with hematoxylin/eosin. The yellow arrow indicates melanin aggregation typical of melanomas. **(C)** Image selected from the T_1_-MRI sequence. The yellow arrow points the main melanoma lesion developed in the mouse brain cortex in correspondence of the point of the injection of tumor cells. **(D)** Close-up of the MRI slice that crosses the tumor.

**Figure 1A** shows the sample used in this study. It is a mouse brain affected by melanoma metastases and loaded with NPs. The primary tumor (B16F10 cells) was implanted into the mouse brain cortex; the black arrow in **Figure 1A** indicates the point of injection into the tumor cells. This large cancerous lesion is the main one, other metastases are also present in the posterior side of the brain. Due to the high content of melanin, this specific tumor and its metastases are recognizable by their black color. The image showing the exterior metastases developed in the back side of the brain is available in **Supplementary Information**, **Figure S1**. The chosen animal model mimics the melanoma brain metastases clinically diagnosed in human cases, as described in (3, 50).

NPs were administered intravenously 12 days after the tumor implantation, when the metastases were highly spread. The mouse was sacrificed 1 h after the NPs injection. This time was considered to be sufficient for the NPs to cross the BBB and penetrate into the main tumor and its multiple metastases. XPCT was performed on excised brain, followed by MRI and histology. **Figure 1B** displays an axial HE histological slice of the brain. The large brown dot, indicated by the yellow arrow, is the primary melanoma that developed in the left brain hemisphere. At higher magnification, the histology reveals the presence of tumor cells, clearly visible due to their specific chaotic arrangement and due to the production of black melanin pigmentation. **Figure 1B** confirms that the primary tumor grew at the point of injection of the tumor cells seen in **Figure 1A**. However, this kind of investigation does not provide any evidence about the distribution of the NPs inside the melanoma. Although the NPs tend to form aggregates larger than their original size (3 nm in diameter, see *Materials and Methods*), the NPs clusters are still smaller than the optical resolution of the light microscope used for the histological examination. In a previous study of (51), the NPs aggregates size could be estimated between 200 and 500 nm. The presence of NPs in the primary tumor was monitored by an MRI investigation. An MRI view, selected from the T_1_-weighted MRI-sequence is reported in **Figure 1C**. The yellow arrow shows the same tumor validated by histology. The MRI inspection reveals how deep this primary tumor grew in the brain cortex compared to the injection site. NPs enhanced the tumor shape visualization, since they are T_1_-MRI contrast agents. Thus, the tumor has a white appearance in the color code of the image. A close-up of this MRI view in **Figure 1D** shows the NPs distribution in the tumor lesion, which is not easy to interpret at this spatial resolution (60 µm).

**Figure 2:**
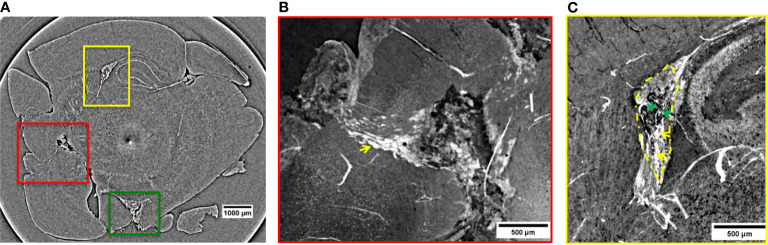
2D XPCT images showing metastases. **(A)** 2D XPCT slice with metastases highlighted within colored rectangles corresponding to **(B, C)** and 3. The dark dot accompanied by a white circle in the center of the slice is an artifact due to the rotation of the sample. **(B)** Volume rendered image of the region of interest indicated by the red rectangle in panel **(A)**. This tilted view shows a large tumor grown after the spreading of the injected tumor cells and, the pathway the NPs reached the tumor. The white small objects pointed out by the yellow arrow are identified as clusters of NPs, while the black dots are melanin pigments typical of melanomas. The image illustrates a complex repartition of NPs, with a major content localized at the edges with the healthy tissue, as indicated by the yellow arrow. **(C)** Volume rendering of the region of interest indicated by the yellow rectangle in panel **(A)**. The image shows a tumor developed in the lateral ventricle. Tumor contour is highlighted by the yellow dotted line. The yellow arrows indicate the tissue areas with a strong accumulation of NPs, while the green arrows point out areas poorer of NP content.

This same tumor was also visualized by XPCT. Thanks to its higher resolution, the XPCT inspection shed light on the NPs repartition (**Figures 2A, B** **)**, offering also insights of the multiple metastases diffused all over the brain (**Figures 2** **–** **5**). Referring to the XPCT gray-scale map, the images show the high-density areas of the brain in white due to the presence of the NPs. The low-density areas in dark gray and black are the transverse vessels, bloodstains, and melanin.

**Figure 2A** is a 2D virtual cross-section of the brain with three regions of interest indicated by colored rectangles. An MRI view of this brain region is displayed in **Figure S6a** of **Supplementary Information**. The volume renderings (see *Materials and Methods*) of the regions under the red and yellow rectangles are displayed in **Figures 2B, C**.

**Figure 3:**
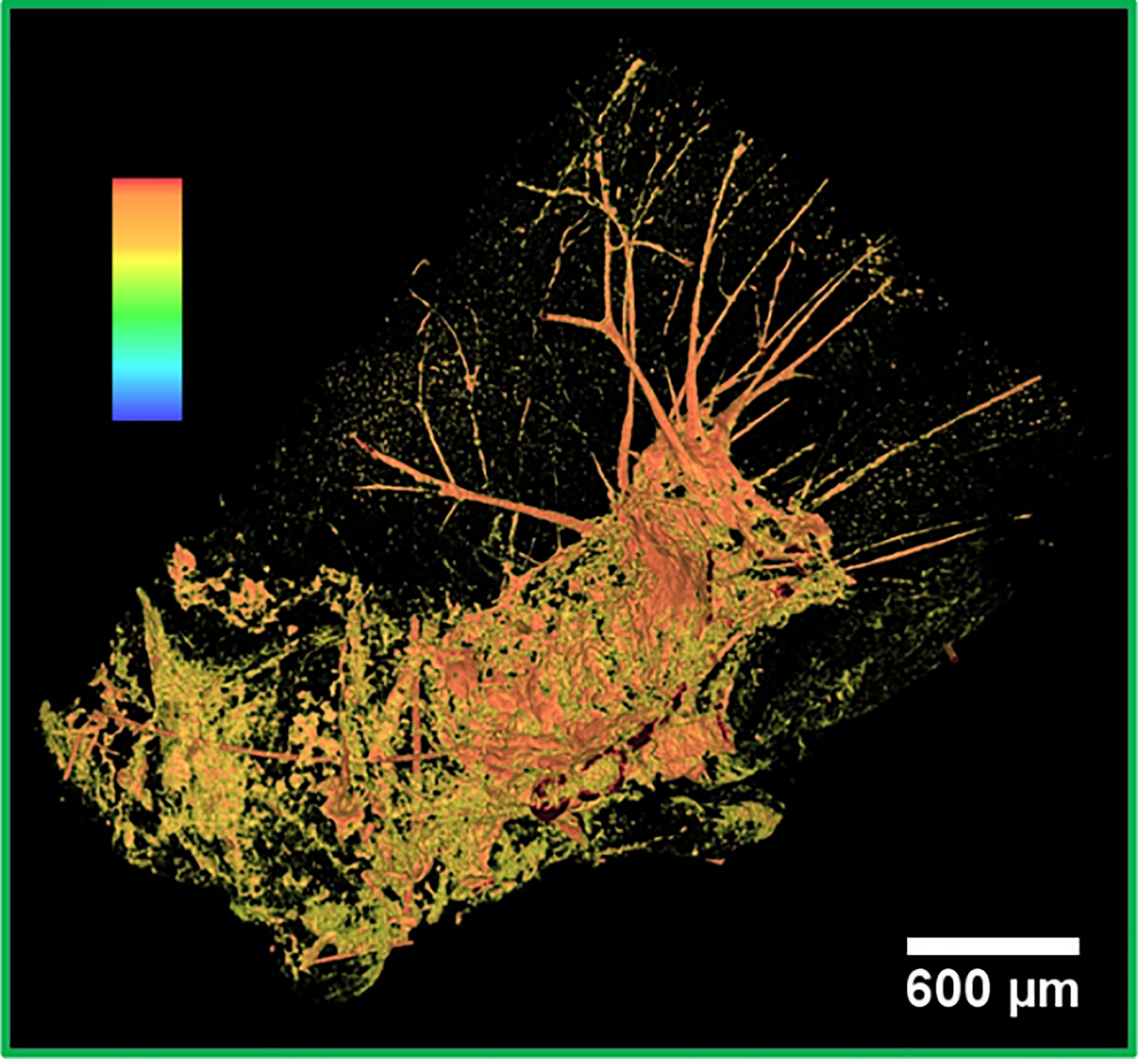
False color 3D image of a metastasis. 3D segmented visualization in false colors of the area under the green box in **Figure 2A**. The highest intensity pixels, displayed in orange, correspond to the densest objects of the image, that is, NPs.

In the red close-up image (**Figure 2B**), it is possible to identify the melanoma cells injected and spread through the cortex and a tumor originated more deeply in the cortex. The tumor is recognizable because of the sudden density change in the cerebral tissue. Unlike the lower-resolution MRI image (**Figures 1C, D** **)**, the XPCT images reveal that NPs (in white) followed a curved trajectory and remained concentrated mostly on the side of this large tumor, as it is indicated by the yellow arrow. Even in the inner tumor core, NPs were visualized. Metastatic melanoma tumors are known to be rich in blood vessels and melanin pigmentation. The large black stains visible at the bounding edges of the tumor might be bloodstains mixed with melanin. The bloodstains are caused by the tendency of melanoma vessels to bleed (52). XPCT demonstrated to be suitable for resolving melanin pigments, and therefore, specific features of this tumor. A sub-cutaneous melanoma was also imaged in this study. An XPCT slice of this control sample is displayed in **Supplementary Information**, **Figure S2**. The yellow rectangle in **Figure 2A** shows a metastasis invading the lateral ventricle. **Figure 2C** represents the volume rendering of this region of interest. The image shows that a certain number of NPs is extravasated from the hemorrhagic flow and deposited in the tumor tissue. NPs were mainly detected in the lower side of the metastasis, as indicated by the yellow arrows (**Figure 2C**). The upper side of the metastasis in **Figure 2C** contains more melanin pigment dots, and only a low content of NPs is visible, as pointed out by the green arrows. In order to characterize the alteration and the density changes induced by multiple melanoma metastases, the anatomy of a healthy brain was also explored by XPCT throughout this study. The control sample was a brain without tumors and has not been injected with NPs (**Supplementary Information**, **Figure S3**). An illustrative XPCT image of the healthy brain tissue is reported in **Supplementary Information**, **Figure S4**. A major focus is placed on the anatomy of the mouse brain ventricle (53), the place where the primary tumor is grown. The morphological differences in this reference sample were useful for identifying the NPs retained in the tumor tissues. The contrast-to-noise ratio (CNR) within the tumor sites depicted in **Figures 2B, C** was estimated to be 6.8 ± 1.1 against the CNR derived from the healthy brain matter, 1.3 ± 0.1. Thus, the significant gain in contrast can be associated to the gadolinium NPs accumulation in tumors. The green box in **Figure 2A** indicates a metastasis which grew in the inferior part of the brain, outside the brain tissue. Here, the CNR was found to be even higher (15.8 ± 0.5) probably due to a denser NPs retention. The 3D representation of the NPs uptake into this metastasis is displayed in **Figure 3**, while its volume rendering with a gray-scale map is reported in **Supplementary Information**, **Figure S5**. The 3D rendered image (**Figure 3**) allows visualizing the complex NPs distribution throughout the tumor at micrometer resolution.

The strong signal in orange corresponds to the highest intensity pixels. Therefore, it can be associated with the NPs accumulation. NPs were mainly detected next to bleeding zones and in the upper part of the tumor. Similar to the other imaged metastases, this metastasis also contains an abundant bleeding. The bloodstains and the melanin correspond to the lowest density pixels rendered in black. The metastasis is surrounded by an irregular and abundant arrangement of the vasculature, as evidenced by the orange network. Apparently, a small amount of NPs did not have enough time to reach the metastasis and remained entrapped in the vessels.

**Figure 4:**
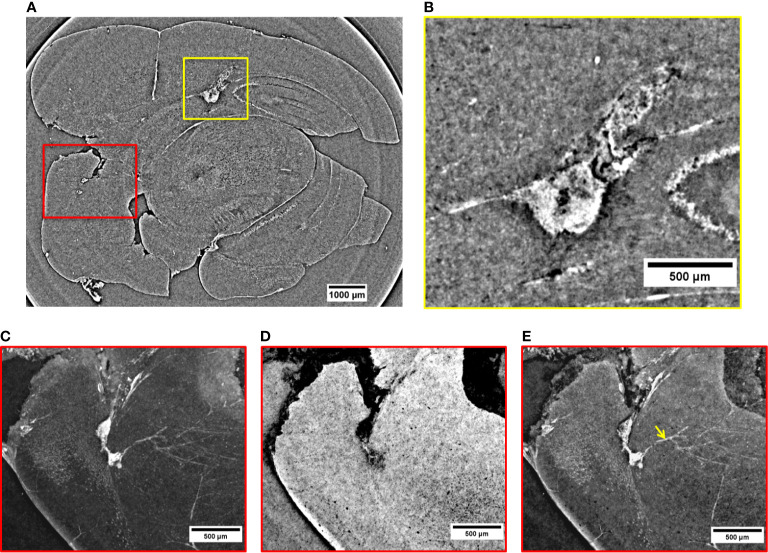
2D XPCT images of tumor lesions. **(A)** 2D XPCT slice showing two tumor lesions in a posterior structure of the brain (red box) and a metastasis developed in the lateral ventricle (yellow box). **(B)** Close-up visualization of the tumor invading the lateral ventricle. **(C)** Projection of maximum intensity values referring to the region of interest under the red box in **(A)**. **(D)** Projection of minimum intensity values referring to the region of interest under the red box in **(A)**. **(E)** Volume rendering of the same area illustrated in **(A, C, D)**. The image displays the accumulation of NPs in the two tumor lesions and in the neighboring vessels (pointed out by the yellow arrow).

In the 2D virtual slice of **Figure 4A**, two regions of interest are highlighted by yellow and red rectangles. An MRI view of this brain region is displayed in **Figure S6b** of **Supplementary Information**. The yellow rectangle indicates another insight of the above-mentioned metastasis attacking the lateral ventricle. Its close-up is illustrated in **Figure 4B**. From this magnified image, it can be noticed that the tumor shape is enhanced thanks to a strong presence of the NPs. They provided a clear contrast between the irregular tumor mass and the surrounding normal brain tissue. The tumor mass is driven by a large amount of blood vessels and melanin (black dots). The irregular shape of the metastasis tends to expand under the cortex by compressing the surrounding healthy tissues. The image reveals a higher density of extravasated NPs close to bloodstains, with smaller clusters of NPs appearing as small dense dots in other parts of the tumor tissue. The red box in **Figure 4A** shows two small metastases originated in a posterior structure of the brain and connected between them through a small vessel. These images allow monitoring the NPs distribution inside each metastasis volume. By applying specific rendering methods (47), data visualization was further enhanced. The NPs distribution was better detectable by the maximum intensity pixel values projection, as depicted in **Figure 4C**. This rendering was achieved through the projection of a neighborhood of 120 slices (see *Materials and Methods*) and, similarly, an image of minimum intensity projection (**Figure 4D**) was produced for the same neighboring of slices. The sum of maximum and minimum values is displayed in **Figure 4E**. The projection of maximum intensity in **Figure 4C** reveals how the NPs are distributed inside the tumor tissue (white area) and how the tangled net of arterioles is connected to the lower metastasis. In addition, the projection of minimum intensity values (**Figure 4D**) shows the brain tissue inhomogeneity due to the tumor. The brain areas attacked by the metastases appear dark with respect to the surrounding brain tissue and are characterized by a relevant concentration of black spots. In both cases, the black areas might be due to a high density of blood vessels and melanin pigments, which are more concentrated close to the external brain surface. Furthermore, the image clearly shows that the two metastases are connected by a blood vessel (longitudinal view of the vessel, **Figure 4D**). This detail was not visible in the single XPCT slice of **Figure 4A**. By reconstructing the volume around this region of interest (**Figure 4E**), it is possible to visualize the co-presence of melanin and NPs in the upper tumor lesion at high resolution. Also, a strong accumulation of NPs becomes apparent in the deeper metastasis perfused by micro-vessels, possibly feeding arterioles. In addition, from **Figure 4E**, it can be noticed that the small white clusters in the vessels (indicated by the yellow arrow) correspond to a small number of NPs entrapped in the circulatory system.

**Figure 5:**
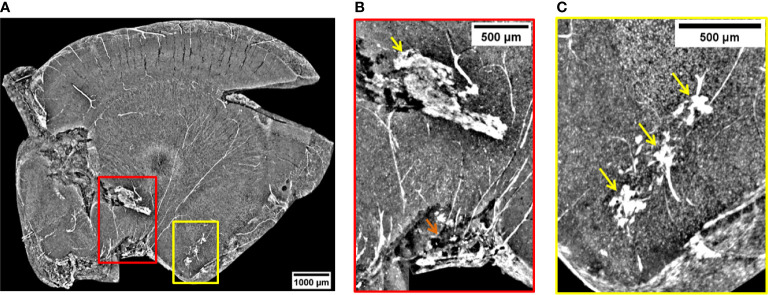
Volume reconstruction of brain bearing metastases. **(A)** 732 µm thick reconstruction of a part of the brain bearing three metastases targeted by NPs. **(B)** Visualization of a metastasis invading the thalamus (yellow arrow) and one developed close to the pituitary gland (orange arrow). **(C)** View on some three metastases in early formation.

**Figure 5A** is a volume rendering (732 µm thickness) showing other three metastases grown in the peripheral regions of the brain (red and yellow rectangles). An MRI view of this brain region is displayed in **Figure S6c** of **Supplementary Information**. The red close-up in **Figure 5B** reveals a malignancy developed in the thalamus (pointed out by the yellow arrow) and another one close to the pituitary gland (orange arrow). Both of them are surrounded by a dense vasculature allowing the transport of a significant number of NPs into the metastases. In particular, the release of NPs close to the pituitary gland is facilitated by a stronger alteration of the BBB (54). Both metastases of **Figure 5B** are a prototype of very well NPs-loaded tumors exhibiting a quasi-homogeneous NPs distribution. The high-density signal detected in the connected vessels demonstrates that a remarkable content of NPs is still stored in the neighboring vasculature and could not fully reach the target before mouse sacrifice. The yellow arrows in the region of interest shown in **Figure 5C** focus on small metastases filled with NPs. The metastases have a reduced size, and they are surrounded by a vessel network.

## Discussion

In this work, XPCT was used for characterizing the NPs distribution inside the melanoma brain metastases. Storage ring based XPCT offers the advantage of evaluating the tissue anatomy with fast measurement scans and at very high resolutions in contrast to tomography laboratory setups. XPCT demonstrated to be a suitable tool for assessing the brain tissue anatomy and the NPs localization without an extensive sample preparation (fluorescent tags, optical agents, stains). Its non-destructive and multi-scale approach allowed imaging the whole brain structure while differentiating anatomical details down to few micrometers. Thanks to its powerful contrast capabilities, it enabled the melanoma metastases recognition by visualizing tissue density changes. In addition, XPCT resolved melanin aggregations that are specific components of this type of tumor. This anatomical feature is normally not detected by other imaging tools, such as MRI. In the study reported here, XPCT revealed to be a powerful tool for tracking the 3D NPs penetration and repartition inside multiple metastases. Thanks to the achieved spatial resolution, 6 µm (effective pixel size 3 µm), it added valuable information about NPs distribution 1 h after injection, a typical time at which irradiation is usually accomplished (55–57). This article shows a new application of the XPCT that opens horizons to the pre-clinical research in the theranostic field. Unlike well-established 2D imaging techniques, XPCT offers the practical advantage of observing the NPs distribution in tissues from multiple points of view. Thanks to the high-resolution, XPCT enabled to simultaneously visualize the NPs and the different brain tissues. This kind of investigation will turn to be useful in the process of NP evaluation. The method will apply for monitoring the nanoparticle clearance, as well as the NPs delivery in tumor sites. Despite MRI being a harmless technique and being considered as a benchmark for the evaluation of the NPs accumulation in tumor, XPCT can complement the MRI findings adding valuable information about the NPs distribution in tissues with higher spatial resolutions. We believe the knowledge of the NPs amount retained in tumors plays a fundamental role in predicting the NPs’ radiosensitizing effects in the further step of tumor irradiation. Since XPCT is a well-consolidated method for characterizing the vascular system (58, 59), it is also capable of revealing if NPs content is still entrapped in the vessels. Thus, it would help in establishing the most effective time for the tumor irradiation, depending on the most homogeneous NPs distribution in tumors. In the case of Gd NPs, more specifically, our findings show information for predicting the therapeutic impact of the NPs during the tumor irradiation phase. Even higher resolution inspections of the NPs distribution might be achieved in the future with X-ray phase contrast nanotomography setups (60) that would allow visualizing more clearly and quantifying the NPs accumulated in tumors. This proof-of-concept experiment demonstrates that XPCT can provide reliable 3D information about NPs localization. This technique can finally complement the classical imaging techniques that are routinely used for evaluating NPs targeting at the pre-clinical state. XPCT can, therefore, find its application as an imaging tool in theranostic research and will deliver valuable information to biologists and clinicians for designing beneficial radiotherapy protocols based on NPs.

## Data Availability Statement

The original contributions presented in the study are included in the article/**Supplementary Material**. Further inquiries can be directed to the corresponding author.

## Ethics Statement

The animal study was reviewed and approved by the French Ministry of Agriculture and carried out in accordance with the official regulation of the French Ministry of Agriculture after approval by the local ethical committee (from Lyon and Grenoble’s Universities, No 380922).

## Author Contributions

EL performed XPCT, processed the images, and wrote the article. LS designed the research, prepared the samples, performed histology, and provided the biomedical interpretation of the images. AC designed the XPCT experiment. EB performed MRI and provided the biomedical interpretation of the images. AB provided assistance as beamline scientist during beamtime. FB and IB contributed to the image processing. MF and LM performed XPCT. IG revised the article. OT and GLD provided NPs. ODLR supervised the work. PZ designed the research and supervised the work. All authors contributed to the article and approved the submitted version.

## Funding

This work was partially funded by the VOXEL project (European Union’s Horizon 2020 research and innovation program under grant agreement no 665207) and COST Action MP1203. The MRI facility IRMaGe is partly funded by the French program “Investissement d’Avenir” run by the French National Research Agency, grant “Infrastructure d’avenir en Biologie Sante” [ANR-11-INBS-0006]. EL and IG gratefully acknowledge the financial support from the Deutsche Forschungsgemeinschaft (DFG, German Research Foundation)-Projektnummer 192346071, SFB 986, project Z2. LS and PZ acknowledge projets interdisciplinaire du CNRS 80 PRIME 2019.

## Conflict of Interest

GLD and OT are employees from NHTherAguix that is developing the AGuIX NPs. GLD, OT, and LS possess shares of this company. ODLR is employed by Imagine Optic.

The remaining authors declare that the research was conducted in the absence of any commercial or financial relationships that could be construed as a potential conflict of interest.
