# Supplementary material for: 3D Spatial Distribution of Nanoparticles in Mice Brain Metastases by X-ray Phase-Contrast Tomography
Source: Front Oncol. 2021 May 25;11:554668. doi: 10.3389/fonc.2021.554668 (PMC8185349; doi:10.3389/fonc.2021.554668)
Supplement: Supplementary file 1 [file DataSheet_1.pdf]

frontiers

# *Supplementary Material*

## 1 SUPPLEMENTARY INFORMATION

An additional illustrative image of the sample bearing melanoma metastases is shown in Fig. S1. The implanted melanoma originated multiple metastases predominantly in empty spaces close to the external brain surface (black areas in the image).

Fig. S2 is a 2D XPCT cropped slice of a sub-cutaneous melanoma. Melanoma is a skin tumor characterized by a high density and chaotic aggregations of melanin pigments (black spots in Fig. S2). As shown in the main text, even melanoma metastases, in minor amount, show similar black aggregation due to melanin presence.

Fig. S3 shows the healthy brain without NPs. This sample was used as control test for characterizing the metastatic regions and identifying NPs in the sick brain. The healthy brain was not perfused post-mortem. In fact, from the image it is possible to recognize the blood vessels.

A XPCT coronal view of the brain (600 μm thickness) is displayed in Fig. S4. The natural contrast provided by XPCT and the high-resolution help in discriminating different anatomic components of the soft tissue. From the image, it is possible to distinguish the grey matter, vessels and nerve fibers. As it can be seen, the tissue density is regular in a healthy organ and there are no evidences of suspicious tumor lesions. If here the focus is shifted on the right ventricle (highlighted by a yellow box in figure), the structure appears normal with well defined boundaries and vessels organized according to a specific pattern, unlike the ventricle invaded by a metastasis (Fig. 2c in the main text).

Fig. S5 shows the volume rendering of the metastasis developed in the inferior part of the brain, outside the brain tissue. The metastasis exhibits a deformed mass pushing the neighboring normal tissue on the front side. The image clearly shows the NPs entrapped in the vessels flowing to the metastasis (see yellow arrows).

Fig. S6 is a combined panel of three different MRI views corresponding to the XPCT slices illustrated in this manuscript.

### 1.1 Figures

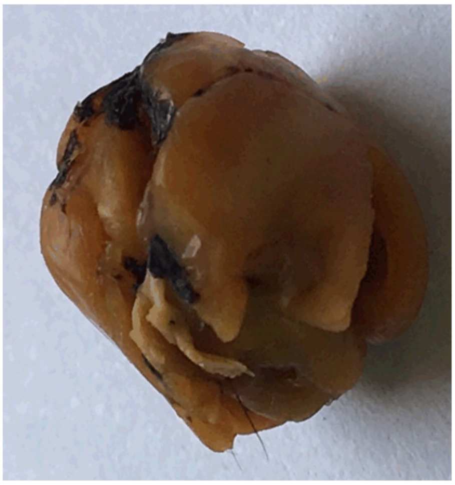

**Figure S1. Melanoma brain metastases.** Image of the posterior side of the mouse brain affected by multiple metastases and injected with NPs.

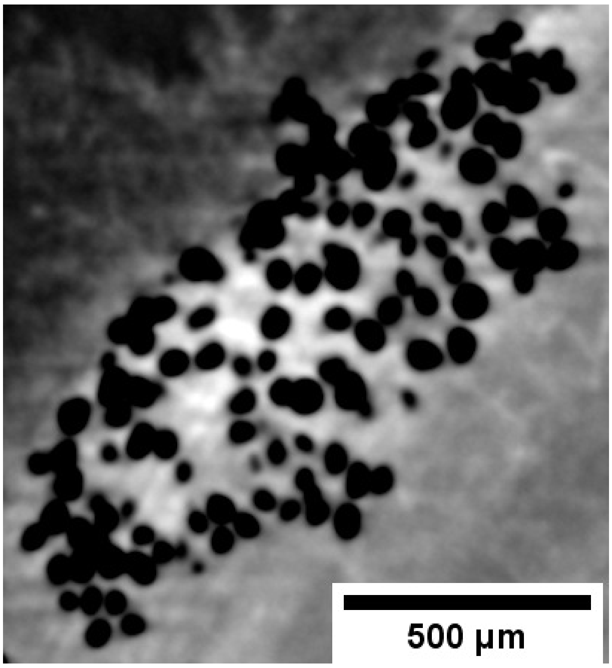


**Figure S2. 2D XPCT slice of a melanoma tumor.** Cropped image of a 2D XPCT cross-section depicting an inner layer of a sub-cutaneous tumor. The black spots are chaotic melanin aggregates.

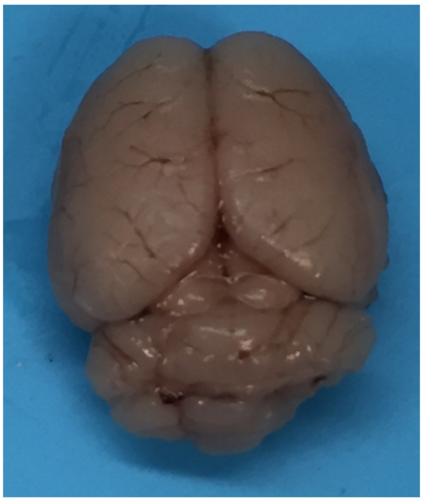

**Figure S3. Healthy mouse brain.** Image of the control mouse brain not perfused.

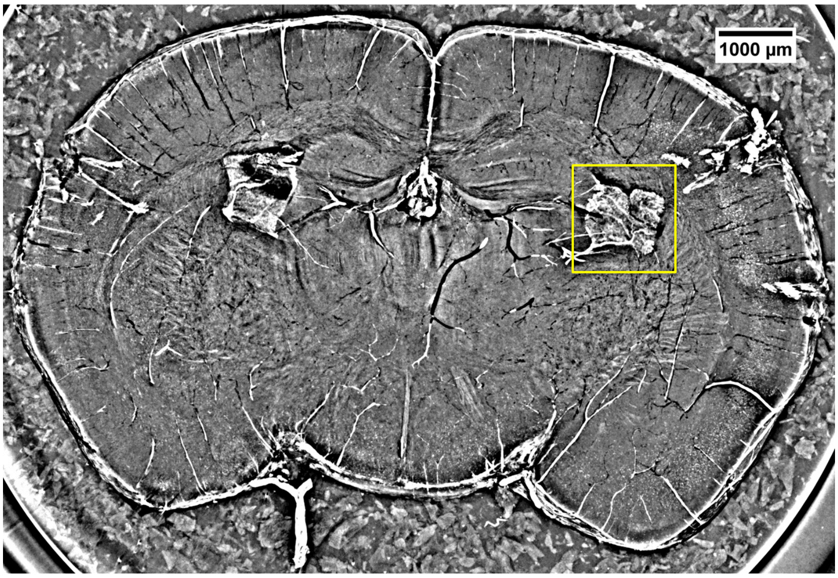


**Figure S4. XPCT volume rendering image of the healthy mouse brain (600 µm thickness)**. The yellow rectangle wants to stress the attention on the healthy lateral ventricle.

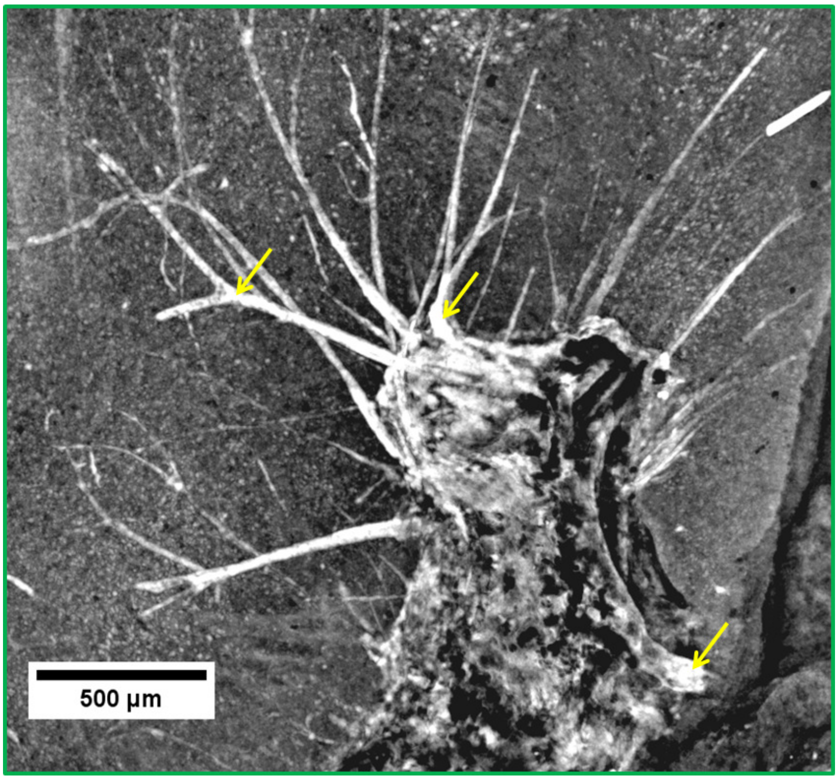


**Figure S5. Volume rendering of a metastasis.** Volume rendering of the metastasis developed in the inferior part of the brain, illustrated under the green box in Fig. 2a of the main manuscript. Yellow arrows show NPs accumulated in the metastasis, especially in the front part and NPs entrapped in the vessels.

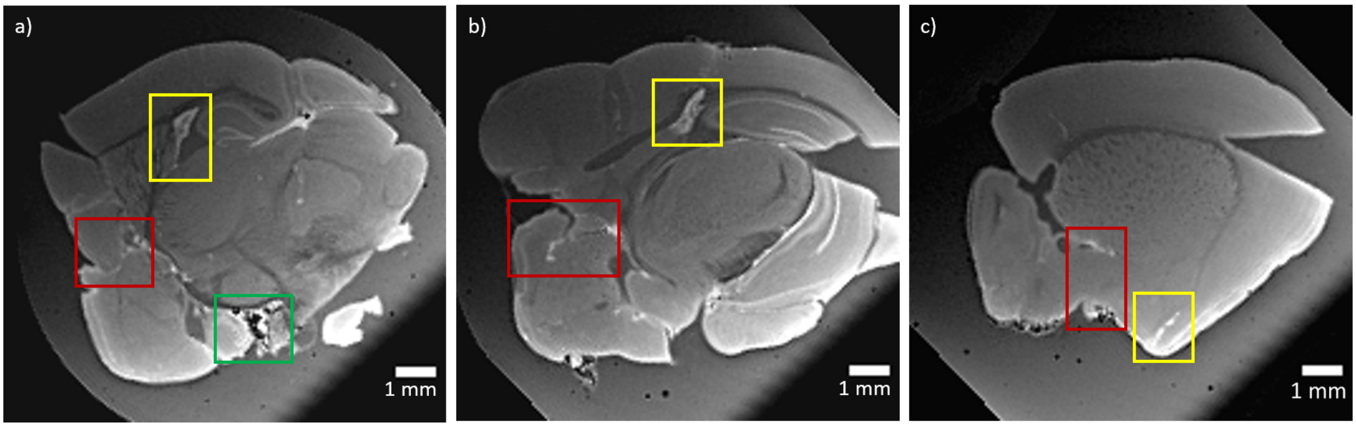


**Figure S6. MRI images.** a) MRI slice corresponding to the 2D XPCT image shown in Figure 2a of the manuscript. The colored rectangles frame the multiple metastases also detected by XPCT with higher resolution . b) MRI slice corresponding to the 2D XPCT image shown in Figure 4a of the manuscript. Similarly to XPCT, the red and yellow rectangles highlight two different tumors filled with NPs. c) MRI slice depicting the metastases developed in the brain thalamus and close to the pituitary gland, as similarly illustrated in Figure 5 of this manuscript.
